# Effects of *Ophiopogon japonicus* By-Products as a Replacement for Alfalfa Meal on Production Performance and Intestinal Health in Meat Rabbits

**DOI:** 10.3390/ani16101538

**Published:** 2026-05-18

**Authors:** Aipeng Mao, Yanbin Chen, Junning Pu, Xiaohong Guo, Shufen Xue, Jing Fu, Jingyi Cai, Gang Jia, Gang Tian

**Affiliations:** Key Laboratory of Animal Disease-Resistance Nutrition of China Ministry of Education, Key Laboratory of Animal Disease-resistant Nutrition of Sichuan Province, Animal Nutrition Institute, Sichuan Agricultural University, Chengdu 611130, China; mao443418199@163.com (A.M.); cyb18793591538@outlook.com (Y.C.);

**Keywords:** carcass traits, growth performance, intestinal health, *O. japonicus* by-products, rabbits

## Abstract

*Ophiopogon japonicus* (*O. japonicus*) by-products contain abundant nutrients and bioactive substances. In this study, we evaluated their effects on production performance and intestinal health in meat rabbits. Firstly, we measured the nutrient levels of *O. japonicus* by-products along with its digestibility in meat rabbits. Then, we replaced dietary alfalfa meal with *O. japonicus* by-products to evaluate its effects on growth performance, carcass traits, apparent digestibility, and intestinal immunity in meat rabbits. The results showed that the effects of *O. japonicus* by-products on growing meat rabbits varied over time. In the early phase (days 1–21), it significantly depressed average daily gain (ADG, *p* < 0.001) and average daily feed intake (ADFI, *p* < 0.001), and increased feed to gain ratio (F:G) (*p* < 0.001). However, in the later phase (days 22–35), a compensatory response emerged, with significantly increased ADG (*p* < 0.001) and reduced ADFI (*p* < 0.01) and F:G (*p* < 0.001). Despite this compensatory growth, the growth performance and feed efficiency for the entire experimental period were not improved. *O. japonicus* by-products decreased carcass weight significantly (*p* < 0.001), but did not significantly affect dressing percentage and meat traits (*p* > 0.05), while it significantly reduced the digestibility of crude protein (CP, *p* < 0.05), increased that of crude fiber (CF, *p* < 0.001), and reduced the activities of amylase (*p* < 0.01) and increased trypsin in cecal contents (*p* < 0.05). Additionally, *O. japonicus* by-products elevated the levels of secretory immunoglobulin A (sIgA) and interleukin-10 (IL-10) in ileal mucosa (*p* < 0.05). They did not significantly affect the cecal microbial community or short chain fatty acid (SCFA) levels (*p* > 0.05). Our research indicated that *O. japonicus* by-products possess a balanced nutritional composition and improve intestinal immunity in rabbit production, making them a viable feed ingredient to partially replace alfalfa meal.

## Simple Summary

In intensive rabbit farming, intestinal diseases are common and cause significant production losses. *Ophiopogon japonicus* (*O. japonicus*) is a traditional Chinese herb whose tuberous root is used in medicine, while the remaining parts (fibrous roots, leaves, flowers, and fruits) are often discarded. To explore a potential solution, this study investigated whether these by-products could be reused as a feed ingredient for meat rabbits. We first analyzed the nutritional content and digestibility, and then fully replaced dietary alfalfa with the processed by-products to evaluate its effects. The results showed that rabbits could digest the by-products effectively. Importantly, it improved gut health by strengthening intestinal immunity and the protective barrier of the gut. However, feeding with by-products also reduced growth performance and feed efficiency in the early stages, leading to lower final body weight. To make it practical for farming, further research is needed to determine the optimal dietary levels to balance its health benefits against its effects on growth.

## 1. Introduction

The tuberous roots of *Ophiopogon japonicus* (*O. japonicus*) are used in traditional Chinese medicine and are rich in nutrients such as carbohydrates, proteins, minerals, amino acids, and vitamin B2 [1,2]. They also contain a variety of bioactive compounds, including steroidal saponins, homoisoflavonoids, and polysaccharides [3]. These compounds have been reported to exhibit a broad spectrum of pharmacological activities, such as cardiovascular protection and anti-inflammatory, antioxidant, immunomodulatory, antitussive, antibacterial, and anti-diabetic effects [1,3,4,5].

Notably, some studies also have identified bioactive compounds in the fibrous roots of *O. japonicus*, such as homoisoflavonoids [6], steroidal glycosides, and saponins [7,8], and the soluble sugar content in fibrous roots is comparable to that in tuberous roots [9]; the levels of saponins and polysaccharides are even higher [10,11,12,13]. Additionally, the aerial parts (flowers, leaves, and fruits) contain higher concentrations of total phenols, total flavonoids, and reducing sugars than both the tuberous and fibrous roots, showing promise for application in health products and pharmaceuticals [14,15]. However, despite this potential, the fibrous roots and aerial parts (*O. japonicus* by-products) remain largely neglected and discarded.

As obligate herbivores and hindgut fermenters [16], diet-related digestive problems are the main cause of production loss in intensive rabbit farming [17,18,19,20]. Given the substantial by-products generated from harvesting *O. japonicus* medicinal materials—whose fibrous roots, flowers, leaves, and fruits are rich in polysaccharides, saponins, and isoflavones [14,15]—we hypothesized that these materials could help enhance intestinal immunity for rabbits. It is reported that polysaccharides from *O. japonicus* fibrous roots have been shown to alleviate gastric ulcers via antioxidant and immunomodulatory pathways [21], and also to mitigate ulcerative colitis by reinforcing the intestinal barrier and modulating gut microbiota (especially increasing *Dubosiella*) [22]. In poultry, enzymatically hydrolyzed and fermented fibrous roots improved disease resistance, immunity, and intestinal health [23]. Similarly, dietary supplementation with polysaccharides from ginseng and *O. japonicus* fibrous roots enhanced antioxidant capacity, immunity, and intestinal barrier function in meat rabbits by increasing microbial diversity, sIgA levels, and tight junction protein expression, thereby improving growth performance and survival [24]. Despite its potential, the use of aerial parts (flowers, leaves, fruits) of *O. japonicus* in animal production has not been reported.

*O. japonicus* by-products comprise the fibrous roots and aerial parts remaining after the harvesting of the medicinal tubers. We hypothesized that these by-products would provide not only fundamental nutrients (such as protein, carbohydrates, minerals, and amino acids) but also bioactive compounds that may enhance intestinal immunity and disease resistance in intensively raised rabbits. To test this point, we first analyzed the nutritional composition and digestibility of the by-products, and then incorporated them into rabbit diets as a replacement for alfalfa meal to evaluate their effects on production performance, nutrient digestion and intestinal health, thereby aiming to develop a novel, low-cost feed ingredient for rabbit production.

## 2. Materials and Methods

### 2.1. Ophiopogon japonicus By-Products

Following collection, the remaining by-products (fibrous roots and aerial parts) were washed, dried, and milled. The resulting material was passed through a 40-mesh sieve and then processed into feed pellets using a granulator.

### 2.2. Nutrient Composition and Digestibility of Ophiopogon japonicus By-Products

#### 2.2.1. Nutrient Composition

Chemical analysis of *O. japonicus* by-products was conducted according to the following procedures. The dry matter (DM), crude protein (CP), Ether extract (EE), starch (ST) crude fiber (CF), neutral detergent fiber (NDF), acid detergent lignin (ADL), calcium (Ca), phosphorus (P)was measured according to the national standards of the People’s Republic of China GB/T 6435-2014 [25], GB/T 6432-2018 [26], GB/T 6433-2006 [27], GB/T 42491-2023 [28], GB/T 6434-2022 [29], GB/T 20806-2022 [30], GB/T 20805-2006 [31], GB/T 6436-2018 [32] and GB/T 6437-2018 [33], respectively, and acid detergent fiber (ADF) was measured by the agricultural industry standard of the People’s Republic of China NY/T 1459-2022 [34]. Gross energy (GE) was measured using an oxygen bomb calorimeter (Parr Instrument Co., Molin, IL, USA). The nitrogen-free extract (NFE) content was calculated with the following formula: NFE (%) = 100% − (CP% + EE% + CF% + Ash%) [35].

#### 2.2.2. Feed Evaluation

A digestibility trial was conducted using 15 healthy, 35-day-old, weaned, mixed-sex New Zealand White rabbits with a similar genetic background and body weight (949.20 ± 1.64 g). Rabbits were individually housed in metabolism cages (L × W × H, 32 cm × 50 cm × 30 cm), with one rabbit per cage. The animals were fed *O. japonicus* by-products as the sole feedstuff twice daily, with free access to feed and water.

Following a 7-day adaptation period to the diet, the feces were collected for 4 consecutive days and immediately treated with 10% HCl for nitrogen fixation. After collection, the daily feces from each rabbit were pooled, mixed, and dried to a constant weight. The dried samples were then ground and passed through a 40-mesh sieve.

The amounts of DM, GE, CP, EE, NFE, CF, NDF, ADF, ADL, Ca, and P in the fecal samples were measured using the same methods as described for *O. japonicus* by-products. The apparent digestibility of each nutrient was calculated using the following formula:

Apparent digestibility (%) = [1 − (Nutrient content in feces/Nutrient content in diet)] × 100

### 2.3. Ophiopogon japonicus By-Products Replacing Alfalfa Meal in Growing Rabbit Diet

#### 2.3.1. Animals, Experimental Design and Diets

We used a completely randomized design for this experiment. A total of 144 healthy, weaned (35-day-old), mixed-sex New Zealand white rabbits with similar genetic background and body weight (895.90 ± 80.69 g) were selected. Rabbits were individually housed in galvanized wire mesh cages (L × W × H, 35 cm × 50 cm × 35 cm), with one rabbit per cage. The rabbits were randomly assigned to one of two dietary treatment groups (*n* = 72 per group): rabbits in the CON group received a basal diet formulated to meet the nutritional requirements of growing rabbits [36], and the TRE group received a diet in which alfalfa meal was completely replaced by *O. japonicus* by-products. This diet was formulated to be isoproteic, isoenergetic, and isofibrous relative to the CON diet. Detailed composition and nutritional levels of the diets are shown in Table S1. All diets were pelleted at the experimental institutionsʹ feed mill and stored in a dark, dry environment prior to the experiment. Rabbits were fed twice daily with ad libitum access to feed and water. The experiment lasted for 35 days, during which animal health and mortality were recorded daily.

#### 2.3.2. Growth Performance

During the experiment, individual body weight and feed intake were recorded weekly. The following growth performance indicators were calculated:

Average daily gain (ADG, g/d) = (Final body weigh − Initial body weight)/Experimental days

Average daily feed intake (ADFI, g/d) = Total feed intake/Experimental days

Feed to gain ratio (F:G) = Total feed intake/(Final body weight − Initial body weight)

#### 2.3.3. Carcass Traits

At the end of the feeding trial, 6 rabbits per group were randomly selected for slaughter by intracardiac puncture with sodium thiopental (75 mg/kg of LW). The dressing percentage was calculated as:

Dressing percentage (%) = carcass weight/live weight × 100

The *Longissimus dorsi* muscle from the right side of each carcass was used to measure meat quality. The initial sample weight (W1) was recorded. The sample was then suspended by a hook through one end (with muscle fibers oriented vertically) and stored at 4 °C for 24 h. After storage, the sample was gently blotted dry and re-weighed (W2). The drip loss was calculated as:

Drip loss (%) = (W1 − W2)/W1 × 100

The pH value of the left hind leg muscle was measured at 45 min and 24 h post-mortem using a portable pH meter (Testo 205, Mörfelden-Walldorf, Germany). Meat color parameters, including lightness (L*), redness (a*), and yellowness (b*), were determined on a freshly cut surface of the same muscle using a colorimeter (CR-400, Konica Minolta, Tokyo, Japan) equipped with a D65 standard illuminant and a 2° standard observer aperture. Three replicate measurements were taken per sample at each time point.

#### 2.3.4. Serum Biochemical Indicators

At the end of the experiment, 6 rabbits per group were randomly selected for blood samples from the heart. Serum was separated by centrifugation at 3500× *g* for 15 min. The concentrations of the following serum biochemical indicators were determined using a 3100 automatic biochemical analyzer (Hitachi, Tokyo, Japan): total protein (TP), albumin (ALB), total cholesterol (TC), triglyceride (TG), glucose (GLU), alkaline phosphatase (ALP), alanine aminotransferase (ALT), aspartate aminotransferase (AST), and blood urea nitrogen (BUN). All assay kits were supplied by Maccura Biotechnology Co., Ltd. (Chengdu, China).

#### 2.3.5. Immune Indicators

At the end of the experiment, 6 rabbits per group were randomly selected. Within 30 min postmortem, a 5 cm segment of the distal ileum was excised and opened longitudinally. The ileal mucosa was gently scraped off using a glass slide, immediately frozen in liquid nitrogen, and stored at −80 °C until analysis. The concentrations of immunoglobulin A (IgA), immunoglobulin G (IgG), immunoglobulin M (IgM), secretory immunoglobulin A (sIgA), interleukin 6 (IL-6), interleukin 10 (IL-10), tumor necrosis factor-*α* (TNF-*α*), and interferon-*γ* (IFN-*γ*) in the ileal mucosa were measured using corresponding enzyme-linked immunosorbent assay (ELISA) kits (Jiangsu Meimian Industrial Co., Ltd., Yancheng, China), following the manufacturersʹ instructions.

#### 2.3.6. Apparent Digestibility

On day 15 of the experiment, 10 rabbits from each group were selected and 4-day fecal samples were collected. The feces were treated with 10% hydrochloric acid for nitrogen fixation, then pooled, mixed, and dried to a constant weight. The dried samples were ground and passed through a 40-mesh sieve. The contents of GE, DM, CP, EE, NFE, CF, NDF, ADF, ADL, Ca, and P in the fecal and diet samples were analyzed using the methods as described previously. Acid insoluble ash (AIA) was measured as an internal digestibility marker. The apparent digestibility of each nutrient was calculated using the following AIA-based formula:

Apparent digestibility (%) = [1 − (AIA in diet/AIA in feces) × (Nutrient in feces/Nutrient in diet)] × 100

#### 2.3.7. Digestive Enzyme Activities

Within 15 min postmortem, the jejunum and cecum were isolated. Approximately 2.0 g of contents from each segment were collected, placed into centrifuge tubes, immediately frozen in liquid nitrogen, and stored at −80 °C until analysis. The contents of the jejunum and cecum were collected post-mortem. The activities of cellulase, pectinase, amylase, and trypsin in these contents were measured using commercial assay kits (Nanjing Jiancheng Bioengineering Institute, Nanjing, China).

#### 2.3.8. Short-Chain Fatty Acids

Approximately 1.5 g of cecal content was weighed into a centrifuge tube, mixed with 4.5 mL of deionized water, and homogenized. The mixture was centrifuged at 5000× *g* for 10 min. Subsequently, 1 mL of the supernatant was transferred to a new tube, mixed with 0.2 mL of metaphosphoric acid solution and 23.3 µL of crotonic acid solution, and allowed to stand for 30 min at 4 °C. After a second centrifugation at 10,000× *g* for 10 min, 0.3 mL of the resulting supernatant was diluted with 0.9 mL of methanol (1:3, *v*/*v*). The diluted sample was centrifuged again and filtered through a 0.22 µm membrane prior to analysis. SCFA quantification was performed using an Agilent 8890 gas chromatograph (Agilent Technologies, Santa Clara, CA, USA) equipped with a flame ionization detector.

#### 2.3.9. Bacterial Community

Genomic DNA (gDNA) was extracted from cecal contents. The full-length 16S rDNA gene was amplified using universal primers (8F: 5′-AGAGTTTGATCATGGCTCAG-3′; 1492R: 5′-CGGTTACCTTGTTACGACTT-3′). Sequencing libraries were prepared using the Nanopore 16S Barcoding Kit (SQK-RAB204,) and Ligation Sequencing Kit (SQK-LSK109, Oxford Nanopore Technologies, Oxford, UK), followed by sequencing on the GridION platform with a MinION Flow Cell. Raw fast5 data were basecalled using Guppy and converted to fastq format. Reads were quality-filtered using NanoFilt (version 2.7.1; minimum Q-score 10; length 1400–1600 bp), and chimeras were removed with UCHIME against the gold database. Taxonomic classification was performed with Kraken2 using the SILVA database (v138), and representative sequences were aligned with MUSCLE (v3.8.31) to construct a phylogenetic tree via FastTree (v2.1.8).

### 2.4. Statistical Analysis

All data were analyzed using SPSS (version 27.0; SPSS Inc., Chicago, IL, USA) with the cage as the experimental unit. Data normality was assessed using the Shapiro–Wilk test, and homogeneity of variance was checked using Levene’s test. When these assumptions were violated, the Mann–Whitney test was used as a non-parametric alternative. Survival rate was analyzed using Fisher’s exact test. Body weight and feed intake (measured weekly) were subjected to repeated-measures ANOVA. The model included treatment, time, and their interaction as fixed effects. When the treatment × time interaction was significant, independent sample t-tests were performed at each time point to compare treatments, with Bonferroni correction applied to adjust for multiple comparisons. Other phase-specific (ADG, ADFI, F:G) and continuous data were analyzed separately for each phase using independent sample t-tests (or Mann–Whitney test if non-normally distributed). For bacterial community, *β*-diversity was assessed based on Bray–Curtis distances, and the significance of differences between groups was tested by permutational multivariate analysis of variance (PERMANOVA) with 999 permutations using the ADONIS algorithm. The differential abundance of microbial taxa was identified using a Mann–Whitney test, and *p*-values were adjusted for multiple testing using the false discovery rate (FDR) method (q < 0.05 considered significant). Statistical significance was set at *p* < 0.05. In the figures and tables, asterisks indicate significance levels (* *p* < 0.05, ** *p* < 0.01, *** *p* < 0.001). Results were visualized using Origin software (version 2025b; OriginLab Corporation, Northampton, MA, USA).

## 3. Results

### 3.1. Nutritional Composition and Apparent Digestibility of Ophiopogon japonicus By-Products

**Figure 1:**
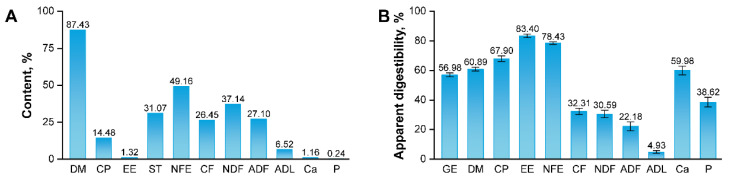
Nutritional composition and apparent digestibility of *Ophiopogon japonicus* by-products. (**A**) Nutritional content of *O. japonicus* by-products. (**B**) Apparent digestibility of *O. japonicus* by-products in growing rabbits. ADF, acid detergent fiber; ADL, acid detergent lignin; Ca, calcium; CF, crude fiber; CP, crude protein; DM, dry matter; EE, ether extract; GE, gross energy, NDF, neutral detergent fiber; NFE, nitrogen-free extract; P, phosphorus; ST, starch.

The nutritional composition of *O. japonicus* by-products is shown in Figure 1A. The contents of GE, DM, CP, EE, ST, NFE, CF, NDF, ADF, ADL, Ca, and P were 17.99 MJ/kg, 87.43%, 14.48%, 1.32%, 31.07%, 49.16%, 26.45%, 37.14%, 27.10%, 6.52%, 1.16%, and 0.24%, respectively. Detailed nutrient and amino acid profiles of different by-product components are provided in Tables S2 and S3. The apparent digestibility of these nutrients and amino acids in growing rabbits is presented in Figure 1B and Table S4.

### 3.2. Effects of Replacing Alfalfa Meal with Ophiopogon japonicus By-Products on Survival Rate and Growth Performance in Growing Rabbits

**Figure 2:**
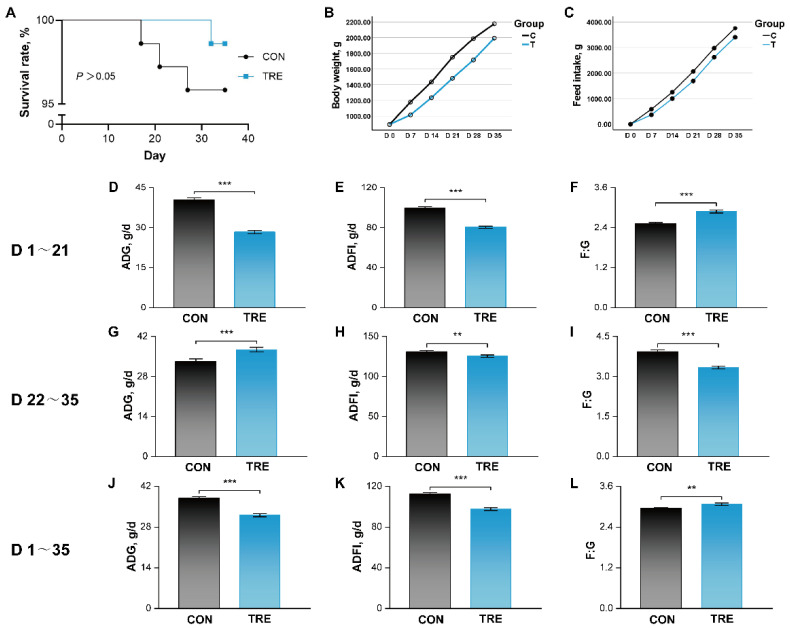
Effects of replacing alfalfa meal with *Ophiopogon japonicus* by-products on survival and growth performance in growing rabbits. (**A**) Survival rate. (**B**) Body weight. (**C**) Feed intake. (**D**–**F**) ADG, ADFI and F:G during days 1–21. (**G**–**I**) ADG, ADFI and F:G during days 22–35. (**J**–**L**) ADG, ADFI and F:G over the entire experimental period (days 1–35). ** and *** indicate *p* <0.01 and 0.001, respectively. ADFI, average daily feed intake; ADG, average daily gain; F:G, feed to gain ratio.

As shown in Figure 2A, the dietary replacement of alfalfa meal with *O. japonicus* by-products numerically increased survival rate, but the difference was not statistically significant (*p* > 0.05). Growth performance data are presented in Figure 2 and Table S5. Repeated-measures ANOVA revealed significant treatment×time interactions for both body weight (F = 39.86, *p* < 0.001) and feed intake (F = 43.05, *p* < 0.001), indicating that the effects of dietary replacement varied over time (Figure 2B,C; Table S5). To explore this interaction further, slice analysis showed that the TRE group had significantly lower body weight and feed intake than the CON group at all time points measured (*p* < 0.001; Table S5). During the early phase (days 1–21), the treatment significantly decreased ADG and ADFI, and increased F:G (*p* < 0.001, Figure 2D–F). In contrast, during the later phase (days 22–35), it increased ADG (*p* < 0.001) and decreased both ADFI (*p* < 0.01) and F:G (*p* < 0.001), indicating improved growth efficiency. Over the entire experimental period (days 1–35), the replacement significantly reduced overall ADG (*p* < 0.001) and ADFI (*p* < 0.001), and increased overall F:G (*p* < 0.01). Consequently, final body weight on day 35 was significantly lower in the TRE group compared to the CON group (*p* < 0.001; Table S5).

### 3.3. Effects of Replacing Alfalfa Meal with Ophiopogon japonicus By-Products on Carcass Traits in Growing Rabbits

We further assessed the impact of *O. japonicus* by-products on carcass traits. As presented in Table 1, the replacement of *O. japonicus* by-products significantly reduced the carcass weight (*p* < 0.05), but it had no effect on dressing percentage or drip loss (*p* > 0.05). Similarly, it did not influence muscle pH or meat color (L*, a*, b*), regardless of post-mortem time (45 min or 24 h, *p* > 0.05).

### 3.4. Effects of Replacing Alfalfa Meal with Ophiopogon japonicus By-Products on Serum Biochemistry Indicators in Growing Rabbits

Serum biochemical analysis revealed that dietary *O. japonicus* by-products significantly reduced GLU levels (*p* < 0.01, Table 2). They had no significant effect on indicators related to nitrogen metabolism (TP, ALB, GLB, BUN), lipid metabolism (TC and TG), or liver enzyme activities (ALP, ALT, AST, *p* > 0.05).

### 3.5. Effects of Replacing Alfalfa Meal with Ophiopogon japonicus By-Products on Ileal Mucosal Immunity in Growing Rabbits

**Figure 3:**
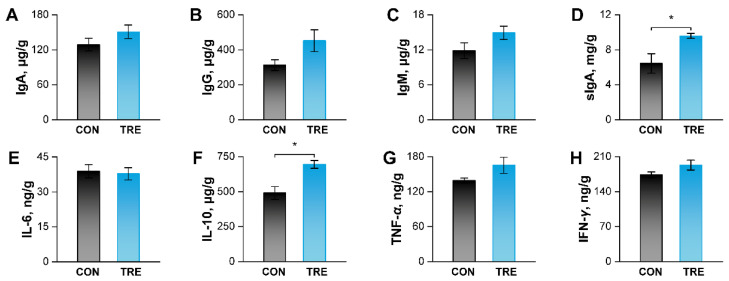
Effects of replacing alfalfa meal with *Ophiopogon japonicus* by-products on ileal mucosal immunity in growing rabbits. (**A**) Concentrations of IgA in ileal mucosa. (**B**) Concentrations of IgG in ileal mucosa. (**C**) Concentrations of IgM in ileal mucosa. (**D**) Concentrations of sIgA in ileal mucosa. (**E**) Concentrations of IL-6 in ileal mucosa. (**F**) Concentrations of IL-10 in ileal mucosa. (**G**) Concentrations of TNF-*α* in ileal mucosa. (**H**) Concentrations of IFN-*γ* in ileal mucosa. * indicate *p* <0.05. IFN-*γ*, interferon-*γ*, IgA, immunoglobulin A; IgG, immunoglobulin G; IgM, immunoglobulin M; IL-6, interleukin-6; IL-10, interleukin-10; sIgA, secretory immunoglobulin A; TNF-*α*, tumor necrosis factor-*α*.

The levels of key immune indicators in the ileal mucosa are shown in Figure 3. Dietary *O. japonicus* by-products significantly increased the concentrations of sIgA and IL-10 (*p* < 0.05). No significant differences were observed for IgA, IgG, IgM, TNF-*α*, or IFN-*γ* (*p* > 0.05), although slight upward trends were observed.

### 3.6. Effects of Replacing Alfalfa Meal with Ophiopogon japonicus By-Products on Apparent Nutrient Digestibility and Digestive Enzyme Activities in Growing Rabbits

**Figure 4:**
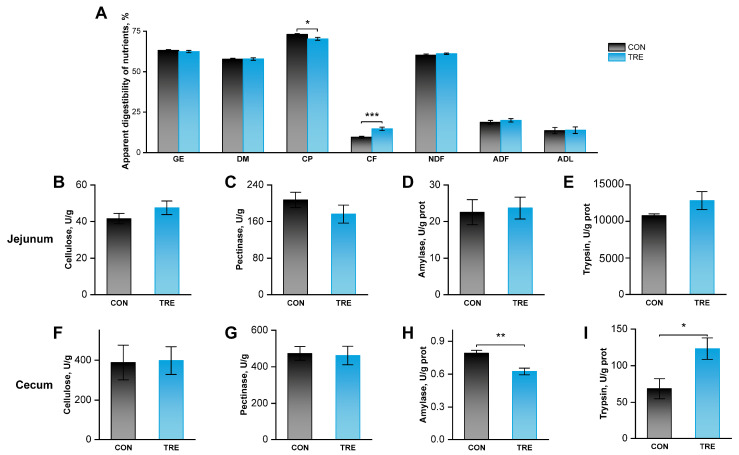
Effects of replacing alfalfa meal with *Ophiopogon japonicus* by-products on apparent nutrient digestibility and digestive enzyme activities in growing rabbits. (**A**) Apparent nutrient digestibility. (**B**–**E**) The activity of cellulose, pectinase, amylase, and trypsin in jejunal contents. (**F**–**I**) The activity of cellulose, pectinase, amylase, trypsin in cecal contents. *, ** and *** indicate *p* <0.05, 0.01 and 0.001, respectively.

To investigate the reasons underlying the altered growth, we assessed apparent nutrient digestibility and digestive enzyme activities. As shown in Figure 4A, *O. japonicus* by-products significantly reduced CP digestibility (*p* < 0.05), but increased CF digestibility (*p* < 0.001). They did not affect the digestibility of GE, DM, NDF, ADF, or ADL (*p* > 0.05). Furthermore, the by-products significantly reduced the activities of amylase (*p* < 0.01) and increased trypsin in cecal contents (*p* < 0.05), while they had no significant effect on the enzymatic activities in jejunal contents (*p* > 0.05).

### 3.7. Effects of Replacing Alfalfa Meal with Ophiopogon japonicus By-Products on Cecal Bacterial Community and Short Chain Fatty Acids in Growing Rabbits

**Figure 5:**
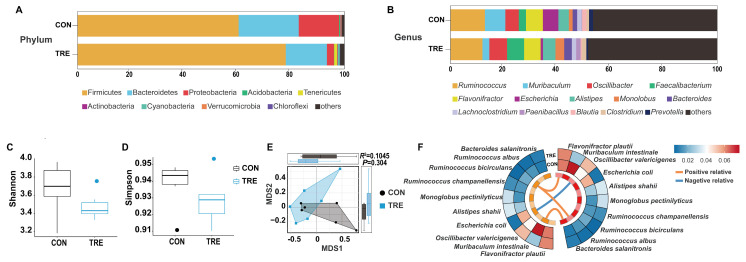
Effects of replacing alfalfa meal with *Ophiopogon japonicus* by-products on cecal bacterial community in growing rabbits. (**A**,**B**) Bacterial community composition at the phylum and genus level. (**C**,**D**) Shannon and Simpson indices of *α* diversity. (**E**) *β* diversity analysis (PERMANOVA on Bray–Curtis distances). (**F**) Correlation of the top 10 species.

Given the observed changes in nutrient digestibility and cecal enzyme activities, we investigated whether the cecal bacterial community and its metabolites were involved. The cecal bacterial composition at the phylum and genus levels is shown in Figure 5A,B. Firmicutes, Bacteroidetes, and Proteobacteria dominated both groups, collectively comprising 98.03% (CON) and 96.11% (TRE), followed by Acidobacteria (0.30%), Actinobacteria (0.24%), Verrucomicrobia (0.17%) and Tenericutes (0.14%) in the CON group, and Tenericutes (1.18%), Cyanobacteria (0.62%), Actinobacteria (0.24%), and Chloroflexi (0.15%) in the TRE group. At the genus level, *Ruminococcus* (CON = 12.89%, TRE = 11.97%) mainly predominated between the two groups, while the CON group was mainly composed of *Muribaculum* (7.68%) and then *Flavonifractor* (6.33), and the TRE group was mainly composed of *Oscillibacter* (6.65%), *Faecalibacterium* (6.39%) and *Flavonifractor* (6.21%). *α* diversity (Shannon, Simpson indices) and *β* diversity (Bray–Curtis PERMANOVA: *R*^2^ = 0.105, *p* = 0.304) did not differ significantly between the groups (*p* > 0.05, Figure 5C–E). Differential abundance of the bacterial community at the genus and species levels was assessed, and no significant differences were detected at either taxonomic level, or in the predicted bacterial functions. (*p* > 0.05, Table S6). Similarly, the concentrations of major bacterial metabolites, SCFAs, did not differ significantly between the two groups (*p* > 0.05, Table 3). Correlation analysis of the top 10 most abundant species revealed that *Ruminococcus bicirculans* was positively correlated with *Ruminococcus albus* and *Flavonifractor plautii*, *Muribaculum intestinale* was positively correlated with *Bacteroides salanitronis*, and *Escherichia coli* was negatively correlated with *Oscillibacter valericigenes* (Figure 5F).

## 4. Discussion

*O. japonicus* by-products contain a variety of nutrients and bioactive compounds. Previous studies indicate that they can enhance growth performance. For instance, enzymatically hydrolyzed and fermented fibrous roots improved weight gain and feed efficiency in broilers [23], while polysaccharides from ginseng and *O. japonicus* fibrous roots reduced diarrhea and mortality in rabbits [24]. Departing from the use of extracts or processed forms, our study directly incorporated the whole by-products into pellets to evaluate the practical feed value. The results demonstrated that *O. japonicus* by-products were nutritionally rich and well utilized by growing rabbits. However, their effects on growth performance varied over time; during the early phase (days 1–21), the by-products significantly decreased ADG and ADFI, while increasing F:G. In contrast, during the later phase (days 22–35), they significantly increased ADG and decreased both ADFI and F:G, indicating a compensatory growth response. Notably, this compensatory gain was insufficient to fully offset the early growth depression, resulting in a significantly lower final body weight in the treated group at the end of the trial. The negative impact on growth during days 1–21 may be attributable to the different fiber components in the by-product diet (Table S1), which reduced voluntary feed intake during adaptation. In contrast, the improved ADG and feed efficiency observed during days 22–35 suggest gradual adaptation of the cecal microbiota and digestive physiology to the novel fiber source.

As hindgut fermenters, rabbits can utilize fibrous agro-industrial by-products, which are considered promising for sustainable production [37]. Beyond growth performance, we assessed carcass traits and meat quality. The results demonstrated that replacing alfalfa with *O. japonicus* by-products decreased carcass weight, but did not alter the efficiency of converting live weight to carcass weight. Furthermore, key meat quality traits (pH and color) remained unchanged.

Serum biochemical analysis serves as a non-invasive tool to assess the physiological and metabolic status of animals, with diet being a key influencing factor [38]. In this study, *O. japonicus* by-products did not significantly alter indicators for nitrogen metabolism (TP, ALB, GLB, BUN), lipid profile (TC, TG), or enzyme activities (ALP, ALT, AST). However, they reduced GLU levels, and this effect may be associated with the well-documented anti-diabetic properties of *Ophiopogon* polysaccharides, which are known to improve insulin sensitivity and glucose metabolism [39,40,41].

The analysis of ileal immunity revealed that *O. japonicus* by-products significantly elevated the levels of sIgA and IL-10. sIgA serves as the first line of defense in protecting the intestinal epithelium from enteric toxins and pathogenic microorganisms. It acts primarily through immune exclusion, which involves blocking pathogen access to epithelial receptors, entrapping them within the mucus layer, and facilitating their clearance via peristalsis and mucociliary activity [42]. Beyond this barrier function, sIgA also participates in immune regulation through several key mechanisms: it facilitates the sampling of antigen–sIgA complexes by microfold cells, engages in close interactions with dendritic cells in Peyerʹs patches, downregulates inflammatory responses, and modulates the responsiveness of both epithelial cells and dendritic cells [43]. IL-10 is an important immunoregulatory cytokine that acts to suppress and terminate inflammatory immune responses, largely through the inhibition of monocyte and macrophage activation [44]. Disruption of the interactions of IL-10 with its receptors (IL-10RA and IL-10RB) and *α*-2-macroglobulin may lead to enhanced inflammation, which could promote tumor growth [45]. In our study, the increase in sIgA and IL-10 suggests that the by-products enhance the intestinal barrier’s first line of defense and promote anti-inflammatory response. In addition, the levels of IgA, IgG, IgM, TNF-*α*, and IFN-*γ* showed upward trends, but the increases were not statistically significant. Collectively, these changes might still contribute to an overall enhancement of intestinal health.

To elucidate the reasons underlying the altered growth performance, we further examined nutrient digestibility and the cecal bacterial community. The results showed that *O. japonicus* by-products significantly reduced CP digestibility and increased CF digestibility. The decrease in CP digestibility may partly explain the poor growth performance. Fiber digestibility is largely mediated by the cecal microbiota, where we also observed significantly altered activities of amylase and trypsin. However, the cecal bacterial community remained unchanged at both the genus and species levels, and the concentrations of their major metabolites, SCFAs, did not differ significantly between groups. Taken together, the stable composition of the cecal microbiota and unaltered SCFA levels suggest that the improved fiber digestibility induced by *O. japonicus* by-products was not attributable to the cecal bacterial community. Instead, it may be associated with other mechanisms, such as altered enzymatic activities.

## 5. Conclusions

In summary, dietary inclusion of *O. japonicus* by-products as a complete replacement for alfalfa meal significantly reduced overall growth performance and carcass weight in growing meat rabbits, although dressing percentage and meat traits were not adversely affected. Importantly, the by-products enhanced intestinal mucosal immunity, as evidenced by increased sIgA and IL-10 levels. These findings indicate that *O. japonicus* by-products possess functional potential to improve gut health. However, the severe growth depression observed under the complete replacement regimen makes this practice unacceptable for practical rabbit production. Future research should explore lower inclusion levels or combination strategies (e.g., enzyme supplementation, fermentation) to mitigate the negative growth effects while preserving the intestinal benefits.

## Figures and Tables

**Table 1:** Effects of replacing alfalfa meal with *Ophiopogon japonicus* by-products on carcass traits in growing rabbits.

Items	Treatments		*p* Value ^1^
	CON	TRE	
Carcass weight, g	1190.33 ± 7.52	1117.33 ± 7.69	<0.001
Dressing percentage, %	54.79 ± 0.70	54.85 ± 0.30	0.942
Drip loss, %	6.61 ± 0.27	6.67 ± 0.40	0.904
pH_45min_	6.94 ± 0.03	6.72 ± 0.14	0.155
L*_45min_	53.59 ± 0.95	53.12 ± 0.82	0.714
a*_45min_	8.30 ± 0.88	8.98 ± 0.87	0.600
b*_45min_	10.66 ± 0.52	11.39 ± 0.47	0.323
pH_24h_	5.80 ± 0.06	5.85 ± 0.05	0.525
L*_24h_	55.07 ± 0.94	56.60 ± 1.21	0.340
a*_24h_	10.32 ± 0.98	9.72 ± 0.85	0.655
b*_24h_	14.28 ± 0.52	14.00 ± 0.69	0.756

^1^ *p* value represents the significance level of the difference between CON group and TRE group.

**Table 2:** Effects of replacing alfalfa meal with *Ophiopogon japonicus* by-products on serum biochemistry in growing rabbits.

Items	Treatments		*p* Value ^1^
	CON	TRE	
TP, g/L	56.39 ± 0.90	54.72 ± 2.25	0.505
ALB, g/L	40.68 ± 1.00	38.74 ± 1.00	0.200
GLB, g/L	15.72 ± 1.32	15.98 ± 1.99	0.915
TC, mmol/L	1.76 ± 0.25	2.02 ± 0.17	0.406
TG, mmol/L	1.48 ± 0.03	1.60 ± 0.14	0.432
GLU, mmol/L	9.25 ± 0.32	7.70 ± 0.27	0.006
ALP, U/L	256.25 ± 6.46	294.83 ± 23.43	0.230
ALT, U/L	89.87 ± 13.12	97.08 ± 9.07	0.661
AST, U/L	71.92 ± 13.22	39.83 ± 6.18	0.052
BUN, mmol/L	5.23 ± 0.53	5.70 ± 0.58	0.568

^1^ *p* value represents the significance level of the difference between CON group and TRE group.

**Table 3:** Effects of replacing alfalfa meal with *Ophiopogon japonicus* by-products on short chain fatty acids in growing rabbits.

Items	Treatments		*p* Value ^1^
	CON	TRE	
acetic acid, mg/g	11.62 ± 1.07	11.66 ± 0.61	0.974
propionic acid, mg/g	1.26 ± 0.09	1.01 ± 0.08	0.071
butyric acid, mg/g	2.99 ± 0.53	3.12 ± 0.34	0.845
isobutyric acid, mg/g	0.10 ± 0.02	0.05 ± 0.01	0.060
valeric acid, mg/g	0.27 ± 0.02	0.28 ± 0.05	0.826
isovaleric acid, mg/g	0.05 ± 0.02	0.01 ± 0.00	0.115

^1^ *p* value represents the significance level of difference between the CON group and TRE group.

## Data Availability

The data that support the findings of this study are available from the corresponding author upon reasonable request.

## Supplementary Materials

The following supporting information can be downloaded at https://www.mdpi.com/article/10.3390/ani16101538/s1, Table S1: Composition and nutrient levels of diets (as-fed basis, %); Table S2: Content of nutrients in different parts of *O. japonicus* by-products (DM basis, %); Table S3: Content of amino acid in different parts of *O. japonicus* by-products (DM basis, %); Table S4: The digestibility of the nutrients and amino acids of *O. japonicus* by-products in growing rabbits; Table S5: Effects of replacing alfalfa meal with *Ophiopogon japonicus* by-products on body weight and feed intake in growing rabbits. Table S6: Comparison of the relative abundance of differential genus, species and function prediction between two groups.

## Abbreviations

The following abbreviations are used in this manuscript:

ADF	acid detergent fiber
ADFI	average daily feed intake
ADG	average daily gain
ADL	acid detergent lignin
AIA	acid insoluble ash
ALB	albumin
ALP	alkaline phosphatase
ALT	alanine aminotransferase
AST	aspartate aminotransferase
BUN	blood urea nitrogen
Ca	calcium
CF	crude fiber
CP	crude protein
DM	dry matter
EE	ether extract
F:G	feed to gain ratio
GE	gross energy
GLU	glucose
IFN-*γ*	interferon-*γ*
IgA	immunoglobulin A
IgG	immunoglobulin G
IgM	immunoglobulin M
IL-6	interleukin 6
IL-10	interleukin-10
NDF	neutral detergent fiber
NFE	nitrogen-free extract
*O. japonicus*	*Ophiopogon japonicus*
P	phosphorus
sIgA	secretory immunoglobulin A
SCFA	short chain fatty acids
ST	starch
TC	total cholesterol
TG	triglyceride
TNF-*α*	tumor necrosis factor-*α*
TP	total protein
