# Supplementary material for: Effects of Ophiopogon japonicus By-Products as a Replacement for Alfalfa Meal on Production Performance and Intestinal Health in Meat Rabbits
Source: Animals (Basel). 2026 May 18;16(10):1538. doi: 10.3390/ani16101538 (PMC13203854; doi:10.3390/ani16101538)
Supplement: Supplementary file 1 [file animals-16-01538-s001.zip › Supplemantary files (Ophiopogon japonicus ).pdf]

Table S1 Composition and nutrient levels of diets (as-fed basis, %)

Ingredients	Groups	
	**CON**	**TRE**
Corn	18.06	18.95
Soybean meal	8.11	7.57
Ophiopogonis japonicus by-products	0.00	16.00
Alfalfa meal	16.00	0.00
Wheat bran	18.03	18.97
Rapeseed meal	5.00	5.00
Rice bran	9.08	9.23
Soybean hull	11.00	11.00
Rice bran and hull	9.37	9.10
Wheat middling	2.10	2.10
Soybean oil	1.26	0.00
Premix[1]	1.00	1.00
CaCO3	0.57	0.66
NaCl	0.30	0.30
L-Lys, ≥78.8%	0.04	0.04
DL-Met, ≥99%	0.03	0.01
L-Thr, ≥98.5%	0.05	0.07
Total	100.00	100.00
Nutrient levels[2]		
DE, MJ/kg	10.00	10.00
CP	15.00	15.00
CF	15.50	15.50
NDF	33.09	33.44
ADF	19.28	18.41
ADL	5.42	5.31
Ca	0.60	0.60
P	0.58	0.59
Lys	0.57	0.57
Met	0.22	0.23
Thr	0.43	0.43

[1]premix provided the following per kilogram of the diet: Fe 30 mg, Cu 6 mg, Zn 35 mg, Mn 8 mg, Co 0.3 mg, I 0.4 mg, VA 6000 IU, VD 900 IU, VE 15 IU, VK3 1 mg, VB12 10 μg, Biotin 100 μg, Choline 100 mg, Pyridoxine 0.5 mg, Riboflavin 3 mg, Niacin 35 mg, Pantothenic acid 10 mg.

[2]Nutrient levels were calculated values.

Table S2 Content of nutrients in different parts of *O. japonicus* by-products (DM basis, %)

**Items**	***O. japonicus* by-products**	**Fibrous root**	**Above-ground parts**
GE, MJ·kg-1	17.99	17.83	18.69
DM	87.43	87.41	87.48
CP	14.48	12.54	14.95
EE	1.32	1.22	1.82
ST	31.07	55.42	11.14
NFE	49.16	59.44	43.93
CF	26.45	17.50	31.04
NDF	37.14	33.36	39.74
ADF	27.10	21.82	30.54
ADL	6.52	4.79	7.80
Ca	1.16	1.04	1.13
P	0.24	0.13	0.22

Table S3 Content of amino acid in different parts of *O. japonicus* by-products (DM basis, %)

Items	***O. japonicus* by-products**	**Fibrous root**	**Above-ground parts**
Ala	0.60	0.38	0.77
Arg	1.11	1.68	0.71
Asp	0.98	0.76	1.12
Cys	0.36	0.26	0.42
Glu	1.87	1.29	2.33
Gly	0.52	0.33	0.65
His	0.22	0.17	0.26
Ile	0.42	0.26	0.53
Leu	0.77	0.51	0.95
Lys	0.72	0.46	0.90
Met	0.26	0.16	0.32
Phe	0.44	0.23	0.58
Pro	1.14	0.76	1.50
Ser	0.52	0.41	0.60
Thr	0.47	0.34	0.56
Tyr	0.33	0.25	0.41
Val	0.54	0.36	0.69

Table S4 The digestibility of the nutrients and amino acids of *O. japonicus* by-products in growing rabbit.

Items	Digestibility	Essential amino acid	Digestibility	Non-essential amino acid	Digestibility
GE	56.98±1.38	Arg	86.20±0.82	Ala	68.73±1.75
DM	60.89±1.32	Cys	62.90±1.66	Asp	72.64±1.51
CP	67.90±1.95	His	72.37±1.75	Glu	80.83±1.19
EE	83.40±1.20	Ile	67.91±2.07	Gly	65.01±1.85
NFE	78.43±0.82	Leu	72.46±1.63	Pro	85.27±1.42
CF	32.31±2.05	Ly	66.17±2.03	Ser	70.00±1.48
NDF	30.59±2.51	Met	65.84±1.88	Tyr	67.64±2.28
ADF	22.18±3.09	Phe	68.02±1.94		
ADL	4.93±0.79	Thr	65.18±2.28		
Ca	59.98±2.93	Val	71.24±1.89		
P	38.62±3.25				

Table S5 Effects of replacing alfalfa meal with *Ophiopogon japonicus* by-products on body weight and feed intake in growing rabbits.

Item	Treatments		*P*-value[1]
	CON	TRE	
Body weight, g			
Day 0	896.28±9.44	895.75±9.54	0.987
Day 7	1179.81±16.46	1016.67±12.99	<0.001
Day 14	1434.00±18.63	1237.28±14.92	<0.001
Day 21	1747.40±19.99	1484.03±18.76	<0.001
Day 28	1988.84±20.74	1719.19±23.07	<0.001
Day 35	2178.23±30.79	1995.32±26.27	<0.001
Feed intake, g			
Day 7	584.35±11.22	366.47±8.40	<0.001
Day 14	1253.93±23.11	1000.38±14.63	<0.001
Day 21	2066.26±39.16	1692.54±25.21	<0.001
Day 28	2976.65±60.52	2622.35±35.37	<0.001
Day 35	3758.78±84.35	3403.89±47.00	<0.001

[1] *P*-values are from slice analysis following a significant treatment×time interaction in repeated-measures ANOVA. For body weight: treatment effect F=63.45, $P<0.001$; time effect F=1100.62, $P<0.001$; treatment×time interaction F=39.86, $P<0.001$. For feed intake: treatment effect F=43.05, $P<0.001$; time effect F=1489.76, $P<0.001$; treatment×time interaction F=43.05, $P<0.001$.
